# Murine Models of Nonalcoholic Fatty Liver Disease and Steatohepatitis

**DOI:** 10.1155/2013/237870

**Published:** 2012-12-12

**Authors:** Masashi Ninomiya, Yasuteru Kondo, Tooru Shimosegawa

**Affiliations:** Division of Gastroenterology, School of Medicine, Tohoku University, Sendai 9808574, Japan

## Abstract

In 1980, Ludwig et al. first reported patients of steatohepatitis who lacked a history of excessive alcohol consumption but showed liver histology resembling alcoholic hepatitis and progression to cirrhosis of the liver accompanied by inflammation and fibrosis. The development of nonalcoholic steatohepatitis (NASH) is associated with obesity, diabetes mellitus, insulin resistance, and hyperlipidemia. However, the pathogenesis of NASH remains incomplete. A “multiple-hit” hypothesis for the pathogenesis of NASH based on an animal model has been proposed and remains a foundation for research in this field. We review the important dietary and genetic animal models and discuss the pathogenesis of NASH.

## 1. Introduction

Although, until recently, fatty liver had been thought to take a benign clinical course, it is now known that nonalcoholic fatty liver disease (NAFLD) or NASH may progress to cirrhosis of the liver accompanied by inflammation and fibrosis [1]. It appears that the prevalence of simple steatosis is from 20% to 30% and in NASH from 2% to 3% in adults, and that NAFLD rarely leads to cirrhosis (3%) compared to NASH (up to 30%) [2]. Obesity, diabetes mellitus, insulin resistance, and hyperlipidemia are conditions frequently associated with NASH [3, 4]. 

The liver histology of NASH was confirmed by Brunt and colleagues and the NASH Clinical Research Network [5, 6]. The diagnostic standards include the extent of steatosis, hepatocellular inflammation, and fibrosis. In steatosis, microvesicular and macrovesicular lipid droplets can be revealed in the whole cytoplasm. In contrast to simple steatosis, in steatohepatitis (NASH) there is inflammation with the presence of steatosis, hepatocellular ballooning, and both lobular and portal inflammation with fibrosis. Moreover, pericellular fibrosis with collagen secreted from hepatic stellate cells is a typical formation that can spread to the portal area and lead to portal-portal and portal-central bridging fibrosis and cirrhosis [7].

The development and progression of NAFLD to NASH would occur via a “two-hit” process involving the interaction of genetic and environment factors [8, 9]. In brief, the progression from normal, healthy liver to steatohepatitis is a gradual process involving, first, the advance of obesity and insulin resistance and, next, inflammation [10]. However, this “two-hit” hypothesis remains controversial. In general, about 30% of patients in the spectrum of NAFLD develop NASH, while patients with only steatosis tend to remain stable over time [11].

To understand the cause of NASH, several factors should be considered, and animal models may be useful as well as taking into account the previous reports. In this paper, we describe animal models and discuss the etiology and pathogenesis of NAFLD and NASH (Table 1).

## 2. The Pathogenesis of NASH

 The pathogenesis of NASH remains incompletely understood. The use of animal models has been indispensable to clarifying the mechanisms by which NASH develops. Indeed, Day and James have proposed a “two-hit” hypothesis for the pathogenesis of NASH based on an animal model that remains a foundation for research in this field [8]. However, this “two hit” hypothesis is known to involve interactions between insulin resistance, adipokines, adipose tissue inflammation, and other pathogenetic factors. Particularly, it has been proposed that hepatic steatosis may be the source of many distinct injurious factors rather than simply a “first hit” [12]. For this reason, the classical “two-hit” hypothesis of progression to NASH is now being modified by a “multiple parallel hits” hypothesis [13]. In the “multiple parallel hits” model, the first step is insulin resistance. Hyperinsulinemia, caused by insulin resistance, leads to steatosis by the accumulation of fat in the liver due to the increased inflow of free fatty acids (FFA) or de novo lipogenesis. Furthermore, when adaptive mechanisms for stress tolerance are overwhelmed, lipotoxicity and chronic inflammation activate major hepatic injury via oxidative and inflammatory stress and lipid peroxidation [9]. Consequently, all these processes contribute to the development of steatohepatitis, fibrosis, and cancer.

**Figure 1:**
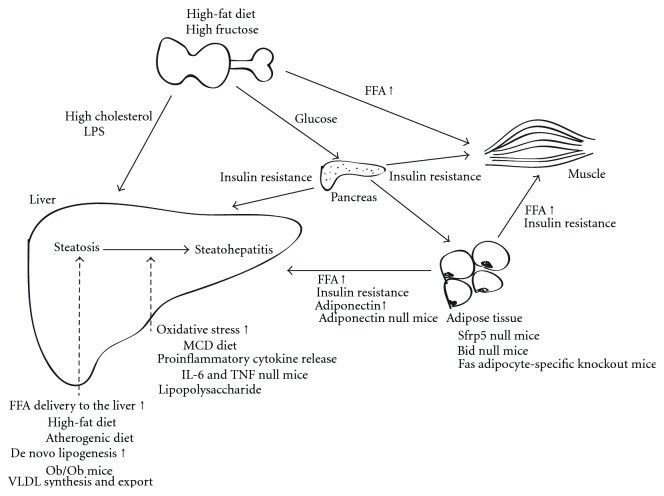
The “multiple parallel hits” hypothesis for the pathogenesis on NASH and the pathophysiological characteristics of animal models. The intake of a high-fat and high-fructose diet and overnutrition lead to metabolic syndrome and obesity. The progression to steatohepatitis in liver is in a stepwise manner involving first the development of fatty changes and, later, hepatic inflammation. Some of the animal models and pathogenic processes are also summarized.

Several mechanisms related to the initial accumulation of fat in the liver have been reported. The impairment of lipid metabolism develops by metabolic syndrome with obesity and excessive oral intake of fats and carbohydrates. The overabundance of FFA also results in fat accumulation. In the field of clinical medicine, protein-calorie malnutrition, jejunoileal bypass, and parenteral nutrition sometimes result in abnormal liver function with triglyceride accumulation by affecting lipoprotein synthesis and causing the inhibition of lipid export from the liver [14, 15]. Excessive triglyceride and the uptake of FFA by the liver induce insulin resistance. Insulin resistance is found in most patients with NAFLD and has been observed in patients who are not obese and have normal glucose tolerance. Moreover, adipokines derived from adipose tissue, such as adiponectin, leptin, and resistin, can be factors in the development of NASH mainly via their effects on insulin resistance [16, 17]. Such multiple factors encompass the interaction of injurious processes including mitochondrial dysfunction and oxidative stress, cytokine-mediated liver injury, altered lipid partitioning and hepatotoxicity mediated by FFA, abnormal intrahepatic cholesterol loading, hyperinsulinemia, hyperleptinemia, and hypoadiponectinemia and apoptosis, which can lead to necroinflammation and, ultimately, fibrosis in the liver (Figure 1) [18, 19]. All of these steps interact in complicated ways to enhance the progression of hepatic lesions through the NAFLD spectrum.

Recently, NASH and simple fatty liver have been suggested to be two independent conditions, and a prospective study showed that the progression of simple fatty liver to NASH is uncommon [20]. Therefore, although simple fatty liver and NASH are considered to be two histological subtypes of NAFLD, these entities may be different not only from a histological, but also from a pathological view point. 

## 3. Dietary Animal Models

### 3.1. Methionine and Choline Deficiency (MCD) Diet

The obese (*ob*) gene was reported to regulate the energy balance in mouse by Zhang et al. Leptin gene deficiency (*ob/ob*) leads to obesity and type II diabetes, which resembles morbid obesity in humans, including NAFLD [21]. A similar clinical condition has been described in *db/db* mouse, which lacks the leptin receptor. Hepatic steatosis and the early stage of inflammatory changes in the liver have been shown in both of these strains of mice, but the MCD diet is required to increase the serum alanine aminotransferase (ALT) levels and develop prominent steatosis in zone 3 (pericentral) and the subsequent necroinflammation which are similar to what occurs in human NASH [22, 23]. The MCD diet promotes intrahepatic lipid accumulation by increasing fatty acid uptake and decreasing very low density lipoprotein (VLDL) production. The expression of triglyceride synthesis-related genes is downregulated [24]. Activated macrophages infiltrate into the liver of MCD-fed mice. The activation of nuclear factor-*κ*B (NF-*κ*B) has been demonstrated together with concomitant increases in the induction of proinflammatory intercellular adhesion molecule-1 (ICAM-1), cyclooxygenase-2 (COX-2), interleukin 6 (IL-6), transforming growth factor-*β* (TGF-*β*), and tumor necrosis factor (TNF) mRNA [25–27]. Although the MCD diet is considered to produce a NASH model, actually the plasma triglyceride and cholesterol levels are decreased, and the metabolic profile is the opposite of that seen in NASH patients. That is to say, serum adiponectin is increased and the insulin, and glucose levels are decreased [28, 29].

### 3.2. Choline-Deficient, L-Amino Acid Defined (CDAA) Diet

The CDAA diet has been used to produce rodent models of steatosis and liver cell death. The serum aspartate and alanine aminotransferase levels are elevated, but CDAA diet rats do not grow fat or show increased insulin resistance [30].

### 3.3. Atherogenic Diet

Matsuzawa et al. fed mice an atherogenic (Ath) diet containg 1.25% cholesterol and 0.5% cholate and reported the progression of steatosis, inflammation, and fibrosis in the liver [31]. Concerning the etiology of the progression to steatohepatitis, it was confirmed that free cholesterol loading caused TNF- and Fas-induction [32]. The mice became obese and showed increased levels of triglycerides and muscle insulin resistance [33]. The hepatocellular ballooning, a characteristic histopathological finding in human NASH, was observed in the mouse livers. Furthermore, gene expression analysis revealed that the Ath diet upregulated the hepatic expression levels of genes for fatty acid synthesis, oxidative stress, inflammation, and fibrogenesis, and down-regulated those of antioxidant enzymes [31].

### 3.4. High-Fat Diet

Animal models fed a high-fat diet, in which the majority of caloric intake is from fat, show metabolic syndrome with fatty liver formation and insulin resistance. A rat model fed a high-fat diet (71% of energy from fat, 11% from carbohydrates, and 18% from protein) develops mild steatosis. Several effects such as insulin resistance, elevation of serum insulin, hepatic lipid accumulation, oxidative stress, and abnormal mitochondria have been reported [34]. The rats became obese and showed abnormal aminotransferase activity similar to that in human NASH. Moreover, immunoblot analysis showed that the expression of CYP2E1 was increased, whereas PPAR-*α* was reduced [35].

### 3.5. Fructose

The increased consumption of high fructose, primarily in the form of soft drinks, is reported to be a risk factor for the development of NAFLD in human. Ingestion of fructose promotes de novo lipogenesis, ATP depletion, and insulin resistance [36, 37]. Mice fed with 30% fructose water showed high levels of hepatic triglycerides and a marked increase in steatosis and weight. Further investigation has shown that dietary fructose intake promotes gut-derived endotoxemia in the portal blood and then the activation of Kupffer cells and hepatic inflammation. Of note, Spruss et al. reported that the mice model of fructose-induced NAFLD is associated with intestinal bacterial overgrowth and increased intestinal permeability, leading to an endotoxin-dependent activation of hepatic Kupffer cells. So, Toll-like receptor 4 (TLR4) knockout mice show decreased levels of steatohepatitis, suggesting that fructose overconsumption can cause hepatic damage [38].

### 3.6. Trans Fatty Acid (TFA) Diet Mice

 Trans fatty acid (TFA), primarily found in partially hydrogenated vegetable oils, is used by the food industry to make products more stable and robust. Its intake is known as a high risk factor for cardiovascular disease, insulin resistance, and obesity [39, 40]. We prepared a low-fat diet (LF 11.8% fat/total nutrition) and high-fat diet (HF 63.6% fat/total nutrition) made of either natural canola oil as the control oil (LF-C and HF-C) or partially hydrogenated canola oil as the TFA-rich oil (28.5% TFA/total fat, LF-T, and HF-T), respectively. Four groups of mice were fed these diets for 24 weeks. 

Concerning the physiological characteristics, the body weight was increased in HF-fed mice, compared to LF-fed mice and HF-T-fed mice weighed 1.3-fold more than HF-C-fed mice. Some serum markers were elevated in the HF-T group compared to the HF-C group, particularly alanineaminotransferase, triglyceride, total cholesterol, (V)LDL-cholesterol, FFA, and leptin were significantly increased. As for the control oil-fed mice, total cholesterol, HDL cholesterol, (V)LDL cholesterol, and adiponectin were lower, whereas plasma leptin was higher in HF-C-fed than in LF-C-fed mice. In contrast, no significant difference was found between LF-C-fed and LF-T-fed mice.

**Figure 2:**
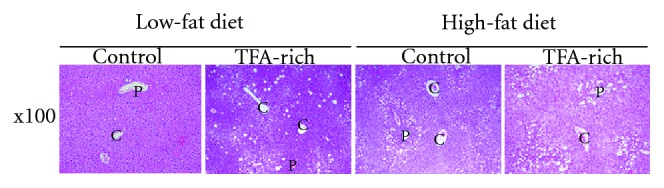
Representative liver histology stained with hematoxylin and eosin stains. The C indicates the central vein, and the P indicates the portal vein.

Few lipid droplets appeared in the LF-fed mice liver, while there were abundant, large lipid droplets in HF-C-fed mouse livers. However, the livers of HF-T-fed mice were characterized by foamy, prominent microvesicular steatosis. Marked, small lipid droplets that surrounded the hepatocytes, inflammation and ballooning degeneration and neutrophil infiltration were expanded in the liver tissue (Figure 2). Moreover, an early fibrosis marker, collagen type1, the *α*1 mRNA level and lipid accumulation in the livers of HF-T-fed mice were increased. An in vitro study demonstrated that TFA intake increased TNF-*α* production and intensified the phagocytotic ability of Kupffer cells [41]. 

In summary, TFA-rich oil intake, in addition to a high fat diet, induces severe steatosis and liver injury.

## 4. Genetic Models

### 4.1. Adiponectin Null Mice

Obesity is one of the risk factors for NAFLD. One cause of obesity is characterized by adipocyte hypertrophy. Adipose tissue secretes many biologically active adipokines. The adipocytokines, such as adiponectin, are decreased in obese adolescents [42]. Hepatic stellate cells (HSCs) play a pivotal role in liver fibrosis [43]. Because adiponectin might suppress the proliferation and migration of HSCs and have biological significance in liver fibrosis, Kamada et al. tried to determine the role of adiponectin using adiponectin null mice and an adenovirus-mediated adiponectin expression system. When mice were administered carbon tetrachloride to cause liver damage, the knockout mice showed liver fibrosis with increased expression of TGF-*β* 1 and connective tissue growth factor, while adenovirus-mediated adiponectin expression mice showed lower levels of liver fibrosis [44]. Two adiponectin receptors (adipoR1 and adipoR2) have been shown in various tissues, and adipoR2 is present predominantly in the liver [45]. Tomita et al. reported that adiponectin receptor AdipoR2 signaling in hepatocytes regulated the steatohepatitis progression by changing the peroxisome proliferator-activated receptor *α* (PPAR-*α*) activity and reactive oxygen species (ROS) accumulation [46].

### 4.2. Adipocyte-Mediated Wnt Signaling: Secreted Frizzled-Related Protein 5 (Sfrp5) knockout Mice

Adipose tissue releases adipokines, such as tumor necrosis factor *α* (TNF*α*), interleukine-6 (IL-6), and leptin. Many of them promote inflammation and disrupt glucose homeostasis [47]. Sfrp5, a protein linked to the Wnt signaling pathway in adipose tissue, is an anti-inflammatory adipokine expressed at higher levels in white adipose tissue [48]. Adenovirus-mediated deliverly of Sfrp5 knockout mice fed a high-fat and sucrose diet became obese and developed insulin resistance and hepatic steatosis. The development of insulin resistance is linked to macrophage-mediated inflammation. And activated macrophages, which accumulate in adipose tissue, are associated with the production of proinflammatory adipocytokines including TNF-*α* and IL-6. Moreover, the c-Jun N-terminal kinase signaling pathway, a downstream target of the noncanonical Wnt signaling, is activated [49].

### 4.3. Adipocyte Apoptosis: BH3 Interacting Domain Death Agonist (Bid) Null Mice

Adipocyte necrotic cell death with macrophage infiltration has been described in both obese humans and mice [50, 51]. The protein Bid leads to apoptosis, regulating the signals to a cell death receptor known as Fas. However, Bid-deficient hepatocytes are resistant to Fas-mediated apoptosis [52, 53]. In Bid null mice, macrophage infiltration in adipose tissue was prevented, which protected against the development of systemic insulin resistance and hepatic steatosis associated with high-fat diet-induced obesity [54]. 

### 4.4. Fas Adipocyte-Specific Knockout Mice

Fas receptor, also known as CD95, a mediator of apoptosis, can regulate inflammatory pathways in several tissues. Fas expression was remarkably increased in the adipocytes of mouse models of obesity and insulin resistance. Mice with selective deletion of Fas were spared the deterioration of glucose homeostasis, insulin resistance, and hepatic steatosis that would otherwise be induced by a high-fat diet. Fas can also regulate inflammation, and the reduced adipocyte, IL6, CD11b, MCP1, and resistin mRNA levels were found in Fas knockout mice, while the levels of noninflammatory IL10 and arginase 1 were increased [55].

### 4.5. IL-6 and TNF Null Mice

The tumor-promoting cytokines IL-6 and TNF caused by obesity are involved in the development of hepatic inflammation and steatosis. IL-6 and TNF null mice have decreased production of hepatic lipid and macrophage infiltration when fed a high-fat diet, resulting in the suppression of steatohepatitis and hepatocellular carcinoma (HCC) formation [56]. This mouse model supports the hypothesis that cytokines generated by adipose tissue can lead to insulin resistance, hepatic inflammation, and steatosis. Moreover, HCC is generated under inflammation and fatty liver in obesity.

### 4.6. Subacute “Inflammation” by Low-Level Activation of NF-*κ*B in the Liver of Transgenic Mice, Designated LIKK Mice

NF-*κ*B signaling regulates a proinflammatory stage that controls the production of a host of inflammatory markers and cytokines, such as CRP, PAI-1, IL-6, TNF-*α*, and IL-1*β*. I*κ*B kinase IKK-*β* inhibits NF-*κ*B activity. LIKK mice showed low level of activation of NF-*κ*B in the livers by selectively expressing constitutively active IKK-*β* in hepatocytes [57]. Moreover, the genetic overexpression of IKK-*β* led to increased glucose levels and hepatic and systemic insulin resistance and increased hepatic production of proinflammatory cytokines. The mice had normal overall appearance in body weight, and their livers were histologically normal [57].

### 4.7. NF-*κ*B Essential Modulator Gene (NEMO^L-KO^) Mice

Obesity results in a chronic inflammatory state, and adipose tissue releases proinflammatory cytokines such as TNF and IL-6. These cytokines lead to insulin resistance in skeletal muscle, liver, and adipose cells. Particularly, TNF-stimulated activation of the JNK and NF-*κ*B signaling pathways has been reported to inhibit insulin action [58]. TNF binding induces the activation of the IKK-1 and -2 kinases that are released from NEMO, thereby regulating the phosphorylation and degradation of I-*κ*Bs liberated by the transcription factor NF-*κ*B [58, 59]. First, Luedde et al. reported that mice lacking NEMO in liver parenchymal cells spontaneously develop chronic hepatitis [60]. Then, glucose metabolism was analyzed in NEMO^L-KO^ mice exposed to a high-fat diet (HFD). These animals are protected from the development of obesity-associated insulin resistance. The authors have reported that hepatic NEMO deficiency with HFD develops in liver steatosis and liver tumorigenesis [61]. 

### 4.8. JNK1^−/−^ Mice

The c-Jun amino-terminal kinases (JNKs) that determine the biological outcome of TNF stimulation can interfere with the action of insulin [62]. Increased JNK activity can develop insulin resistance, and JNK1^−/−^ mice have improved insulin sensitivity and enhanced insulin receptor signaling during obesity [63]. This suggests that JNK may have a role in developing steatosis. Indeed, Schattenberg et al. have shown that JNK1^−/−^ mice fed the MCD diet have significantly reduced steatohepatitis compared with wild-type mice [64].

## 5. Conclusion

This paper has described murine dietary and genetic models of NAFLD, NASH, and steatohepatitis. A number of animal models have been reported, and a variety of factors have been shown to induce fatty liver formation. These factors can induce systemic changes, steatosis, hepatic inflammation, and fibrosis. We cannot elucidate the pathogenesis of NASH using only one type of animal model. Steatosis should be considered as having a key role in the underlying disease process, but evidence has recently suggested that free fatty acids and their metabolites may be one of the major factors in the pathogenesis of NASH because their metabolites are highly toxic to the liver. The factors that potentiate the progression from simple steatosis to NASH, some of which we have discussed, should be the focus of study.

## Figures and Tables

**Table 1:** Dietary models of nonalcoholic fatty liver disease.

Model	Obesity	Insulin resistance	Steatohepatitis	Hepatic fibrosis	Elevated AST and ALT
MCD diet	Weight loss	Hepatic insulin resistance	Yes	Yes	Yes
Choline-deficient, L-amino acid defined (CDAA) diet	Weight loss	No	Yes	Yes	Yes
Atherogenic diet	—∗	Hepatic insulin resistance	Yes	Yes	—
High-fat diet	Weight gain	Yes	Yes	Slight	—
Fructose diet	—	Yes	No	No	Yes
Adiponectin null	Weight gain	Yes	Yes	Yes	Yes
Sfrp5 knockout	Weight gain	Yes	Yes	—	—
Bid null	Weight gain	Yes	Yes	—	—
Fas adipocyte-specific knockout	Weight gain	Yes	No	—	—
IL-6 and TNF null	—	—	No	—	—
LIKK	No change	Yes	No	No	No
NEMO^L-KO^	Weight gain	Yes	Yes	—	—
JNK1^−/−^	Weight gain	Yes	No (only steatosis)	No	—

∗Indicates no data.
